# A Lane-Level LBS System for Vehicle Network with High-Precision BDS/GPS Positioning

**DOI:** 10.1155/2015/531321

**Published:** 2015-02-08

**Authors:** Chi Guo, Wenfei Guo, Guangyi Cao, Hongbo Dong

**Affiliations:** ^1^Global Navigation Satellite System Research Center, Wuhan University, Wuhan, China; ^2^Computer School, Wuhan University, Wuhan, China

## Abstract

In recent years, research on vehicle network location service has begun to focus on its intelligence and precision. The accuracy of space-time information has become a core factor for vehicle network systems in a mobile environment. However, difficulties persist in vehicle satellite positioning since deficiencies in the provision of high-quality space-time references greatly limit the development and application of vehicle networks. In this paper, we propose a high-precision-based vehicle network location service to solve this problem. The major components of this study include the following: (1) application of wide-area precise positioning technology to the vehicle network system. An adaptive correction message broadcast protocol is designed to satisfy the requirements for large-scale target precise positioning in the mobile Internet environment; (2) development of a concurrence service system with a flexible virtual expansion architecture to guarantee reliable data interaction between vehicles and the background; (3) verification of the positioning precision and service quality in the urban environment. Based on this high-precision positioning service platform, a lane-level location service is designed to solve a typical traffic safety problem.

## 1. Introduction

A vehicle network is a special control network which uses advanced sensor technologies, network technologies, computing technologies, and so forth to realize overall perception of the road and vehicle driving state, traffic fine grained control on every road, providing location services to drivers, and finally the security of road traffic safety and traffic efficiency [1–3]. Due to the importance of the space-time reference to the network, the global navigation satellite system (GNSS) becomes the key component of a location based services (LBS) vehicle network. Limited by positioning precision, most current vehicle network systems can only provide simple services such as macroscopic traffic communication. However, more intelligent vehicle network system becomes the new trend with the development of precise positioning technology, which makes it possible to monitor the lane-level vehicle behavior and tracking. In fact, different applications need different positioning precision. For example, road based navigation used now only needs 5~10 m precisely; lane level monitoring and control need 0.5~1.5 m precisely; and, moreover, unmanned drive needs 0.05 m and even more precise.

As described above, the accuracy of space-time information is actually the core factor in the vehicle network location service in a mobile environment. However, currently in urban settings, common GNSS positioning precision can only reach ±5 − ±10 m, which could only support the road level navigation application. In order to improve the positioning accuracy, the widely used method in surveying and mapping field is ground based augmentation system (GBAS), which uses the precise ephemeris and the precise satellite clock correction to realize precise point positioning (PPP). In China, the construction of the BeiDou system (BDS) as well as its area augmentation system is being finished gradually [4]. However, it is mainly used in surveying and mapping filed, which has a few users compared to the vehicle network. The other difference between the vehicle network and the traditional survey application is that the user of the former is usually a fast speed carrier, which has the requirement of the information broadcasting real time and stability feature.

When the GBAS technology and PPP technology of the GNSS are used in the vehicle network to satisfy the lane-level monitoring and control or other similar accuracy applications, the whole system, especially the augmentation information broadcasting system, should be redesigned. This study demonstrates an application of wide-area precise positioning technology [5, 6] to a vehicle network system, to establish a new kind of vehicle network architecture with uniform and high-quality space-time information. Moreover, based on high-precision positioning, this study develops a lane-level location service to satisfy the requirements for active vehicle safety [7]. Active vehicle safety is a new technology used for forecasting traffic accidents like car collisions with the help of sensors and information network, which highly depends on the high-quality space-time information.

Specific to these features, this study focuses on the following issues.

1. In order to satisfy vehicle users' precise positioning demands related to high speed and large-scale mobile targets in the mobile Internet environment, the vehicle network system is integrated with the wide-area real-time precise point positioning (PPP) [8] service, in which we model the positioning error and design a space-time adaptive correction message broadcast protocol.
2. To guarantee reliable data interaction between the server and the high speed vehicles, a concurrent service system with virtualization and flexible expansion architecture is designed. Firstly, a background communication agency with a virtualization-computing feature is provided. Then, a service with high concurrency and high reliability can be realized through dynamic resource allocation and flexible expansion algorithm.
3. Based on the research results of the BeiDou-Xihe system [9], a vehicle precise positioning experiment was conducted in a series of urban environments, to verify positioning precision and service quality. We also devised a vehicle active safety service, which needs to be built on a precise positioning vehicle network system. This service is applied to the traffic supervised system to help vehicles forecast latent dangers such as car collisions.

## 2. Related Work

A vehicle network combines various technologies, such as the GNSS, wireless communication, and vehicle control. The core of such a network is to rapidly and accurately acquire vehicle information to provide timely and reliable service information. The recent rapid development of the mobile Internet lays the foundation for the development of the vehicle network. The vehicle network platform of various vehicle manufacturers are commercially promoted [10, 11], for example, OnStar of General Motors, G-Book of Toyota, and SYNC of Ford. However, these platforms adopt the telematics system major in the vehicle information service, which provides online navigation and traffic information push services. However, this system fails to truly exert the function of the vehicle network system, because of vehicle positioning precision limitations.

The GNSS precise positioning technology has achieved considerable development in recent years. Through various means such as PPP, satellite positioning precision has improved to a submeter, even to a centimeter level. Wide-area real-time PPP is one of the methods suitable for a vehicle network system. The Internet broadcast corrections made the positioning at the terminal to realize the positioning at the decimeter level. However, this kind of correction transfer adopts the network transport of RTCM via Internet protocol [12] and the data formats widely used are the RTCM, RTCA, and compact measurement record. These formats are not applicable to all vehicle networks because of their large data sizes. Therefore, a specific data broadcast and service system needs to be designed.

At present, many new kinds of vehicle network services have been presented, such as for school bus safety and collision avoidance. Duan and Wang (2013) designed a vehicle network-based school bus safety control system to monitor school buses through a vehicle network and set the school buses as high right-of-way targets [13]. When the school bus stops and lets pupils on or off, the vehicle network sends prewarning information to the surrounding vehicles to avoid any accident from occurring. Liu et al. provided a prewarning model and algorithm for vehicle collisions in the vehicle network environment [14], where the Internet was utilized for the transfer and interaction of driving information (including location, speed, and direction) and introduced the time to collision information to establish an anticollision algorithm. Vehicle network location services have clearly developed in the active safety field. To realize this development, a sufficiently precise positioning reference should be constructed to acquire the precise location and status. Taking precise positioning as the basic service of vehicle networks is an urgent issue that demands resolution.

## 3. System Framework

**Figure 1:**
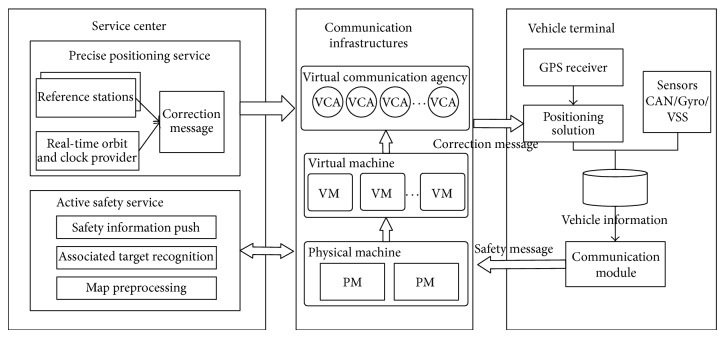
System framework.

The vehicle network system designed in this paper includes a precise positioning and service center, a data communication architecture, and a vehicle terminal, as shown in Figure 1. The positioning service of this system takes advantage of the communication architecture in the vehicle network system. We also created an optimal design for the communication architecture to satisfy the large service range and huge amount of users. The main modules in the system are as follows.

1. Service center: it contains precise positioning service and active safety service. The positioning service module adopts the wide-area real-time PPP technology. The background server receives the real-time precision orbit products provided by the International GNSS Service (IGS) and by the satellite observation data from the reference station and then calculates and extrapolates using this received information. Subsequently, the background server broadcasts correction messages to the users after encoding, which can be used by the vehicle terminal to generate precise positioning. In this paper, we also introduce an active safety service. It is a lane-level dynamic geofence service, which pushes active safety information toward other associated mobile objects according to the precise positioning feedback.
2. Communication infrastructures: a data channel between the terminal and the background is established through the mobile Internet. The communication architecture based on the virtual communication agency (VCA) possesses a flexible expansion and recycling capacity. Through the efficient use of resources and dynamic and adaptive load balancing, concurrent communication within a large-scale vehicle group can be realized.
3. Vehicle terminal: it includes the positioning processing, vehicle information collection, and communication module. The vehicle terminal receives correction messages broadcast by the platform for the positioning processing. The location and vehicle information obtained from the other sensors are then sent to the background by combining the original observations of the GNSS chip.

## 4. Vehicle Network Precise Position Service

### 4.1. Vehicle Precise Positioning Model

Global positioning system (GPS) is widely adopted to determine the vehicle location in most services. The satellite positioning in GPS technologies, however, can be influenced by satellite orbit error, satellite clock error, and ionospheric delay. Consequently, the positioning precision is just ±5 − ±10 m. To meet the requirement of lane level monitoring and control applications, the positioning accuracy should be improved to 0.5~1 m. The current real-time and large-scale precise positioning service technology mainly includes the following.

- Real-time PPP technology: a globally distributed ground GNSS observation station is used to directly calculate the satellite location and satellite clock error, generating an enhanced message that is sent to users. The vehicle terminal can correct the broadcast ephemeris from navigation satellites through this kind of enhanced message and further realizes high-precision positioning independently.
- Network real-time kinematic [15] technology: multiple continuous operational reference systems (CORS) are used in the service area and for the relative positioning of the GPS based on the differential principle. Such systems eliminate the error and influence of terminal positioning.

**Figure 2:**
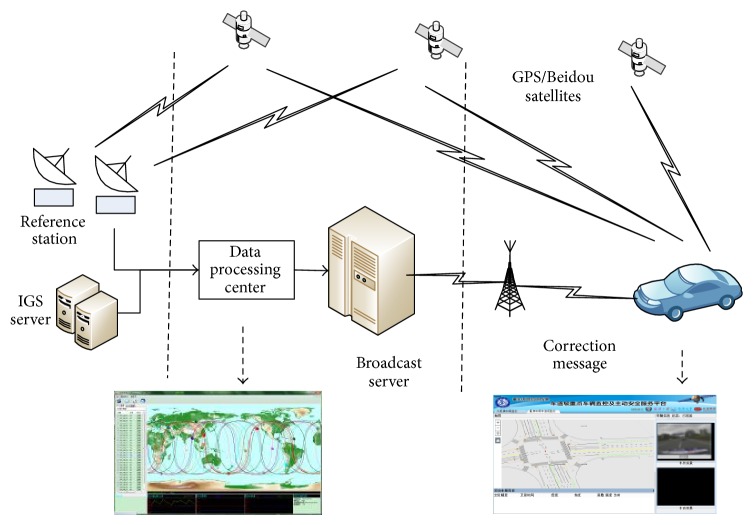
Framework of real-time PPP.

Considering the large population, wide distribution range, and fast movement speed of the targets in a vehicle network, the economic cost and reliable communications are a challenge for a real-time kinematic technology network. Therefore, wide-area real-time PPP technology applied to the vehicle network provided precise location services. The location service model for the wide-area real-time PPP technology as used is shown in Figure 2.

The service model divides into two terminals: the resolve service terminal and the vehicle-positioning terminal. Between the two service models, the function of the resolve service terminal is to broadcast the real-time precise orbit and clock error products and to provide an ionospheres' correction model.

The data source of the server terminal mainly comes from the rapid precise ephemeris released by the IGS forecast. Given the poor precision of the extrapolation of the forecast products, a certain amount of the GPS/BDS CORS needs to be distributed in mainland China. Through real-time and continuous data observation, error is estimated immediately. The resolved corrections are then broadcasted through the Internet. At present, the commonly used communication data formats include TCM, RTCA, SP3, and RENIX. To address the specific disadvantages of the hugely redundant information and large data size of these formats, an adaptive data communication protocol was designed. In addition, the communication data volume was compressed, which enables the service to be applied to the vehicle network system with a large quantity of targets. The terminal uses both real-time correction messages and satellite observations to resolve positioning, which delivers precise positioning coordinates.

### 4.2. Adaptive Correction Message Broadcast Protocol

This section details the adaptive correction broadcast protocol designed according to the vehicle network communication mode and the differences in the correction update rates. This protocol divides the correction content into two parts and adopts different encoding modes and data transfer rates. Simultaneously, the protocol also considers the differences of the user terminals. Thus, the corrections for the different vehicles can be obtained with the fastest speed in different locations and network environments, which can realize the precise positioning rapidly. The specific procedures are as follows.

*Orbit/Clock Error Correction*. The update rate of the satellite orbit clock correction creates demanding requirements. To compress the data volume, we designed a new broadcast protocol, based on the effective bit broadcast mode. In this protocol, it is unnecessary to send the whole ephemeris to the terminals. Actually, we only send the mantissa of the data related to the satellite precise orbit and clock as the correction. Receiving these messages, the terminal combines satellite broadcast ephemeris with the correction to get the satellite precise orbit and clock. The concrete method is as follows.

According to the *N* satellite, at *T*_0_, the precise orbit position and clock error forecast by the system resolve can be noted as *SP*_*T*_0__^*N*^ = {*x*_0_^*s*^, *y*_0_^*s*^, *z*_0_^*s*^, *clk*_0_^*s*^}, while the differential correction can be noted as *Dlt*_*T*_0__^*N*^ = {Δ*x*, Δ*y*, Δ*z*, Δ*clk*}. The terminal receives *Dlt*_*T*_0__^*N*^ at *T*_1_. The satellite broadcast ephemeris is used to resolve the current orbit clock error, which is noted as *P*_*T*_1__^*N*^ = {*x*_1_, *y*_1_, *z*_1_, *clk*_1_}, and the recovered precise orbit position clock error is noted as *SP*_*T*_1__^*N*^ = {*x*_1_^*s*^, *y*_1_^*s*^, *z*_1_^*s*^, *clk*_1_^*s*^}.

A low-bit cut is made on *SP*_*T*_0__^*N*^, and the magnitude portion is reserved for the differential correction and broadcast to users. All the data units are converted to meters. Afterwards, the calculation method of *Dlt*_*T*_0__^*N*^ can be expressed as

(1) $$\begin{array}{c} {Dlt}_{T_{0}}^{N}={SP}_{T_{0}}^{N}-\left\lfloor \frac{{SP}_{T_{0}}^{N}}{100}\right\rfloor \times 100. \end{array}$$

After the user terminal receives the correction *Dlt*_*T*_0__^*N*^, the current broadcast ephemeris from the satellite *P*_*T*_1__^*N*^ is used. The recovered satellite precise orbit and clock error can be expressed as

(2) $$\begin{array}{c} {SP}_{T_{1}}^{N}=P_{T_{1}}^{N}-\left\lfloor \frac{P_{T_{1}}^{N}}{100}\right\rfloor \times 100+{Dlt}_{T_{0}}^{N}. \end{array}$$

The data size of *Dlt*_*T*_0__^*N*^ decreases greatly compared with *SP*_*T*_0__^*N*^. Under normal circumstances, the difference range between *SP*_*T*_0__^*N*^ and *P*_*T*_1__^*N*^ falls within ±30 m. For we need to check the integer part of *SP*_*T*_1__^*N*^ obtained through combined recovery to verify the correctness.

*Ionospheric Correction*. This study divided up the ionosphere model in the form of grids. The grid unit is set according to the distribution of the ionospheric activity. The range is in the area of China. Moreover, ionospheric correction is affected by the positions where satellite signals pass through, so we use bilinear interpolation to calculate a point's ionospheric correction.

The correction mode is as follows: *λ*_0_ and *β*_0_, respectively, represent the starting latitude and longitude of the grid area; Δ*λ* and Δ*β* represent the latitude and longitude spacing, respectively; and *M*∗*N* represents the classified grid quantity (changes with the service area). The ionospheric correction at the grid point is noted as *E*(*m*, *n*); *m* and *n* represent the sequence numbers in the latitude and the longitude directions, respectively. The puncture point coordinate of the satellite signal through the ionosphere is noted as (*λ*′, *β*′), and the interpolation correction is noted as *E*(*λ*′, *β*′).

The ionospheric corrections of the four grid points corresponding to point *V* are noted as *E*(*i*, *j*), *E*(*i* + 1, *j*), *E*(*i*, *j* + 1), and *E*(*i* + 1, *j* + 1). Consider

(3) $$\begin{array}{c} i=\left\lfloor \frac{\lambda^{\prime }-\lambda_{0}}{\Delta \lambda }\right\rfloor ,\;\;j=\left\lfloor \frac{\beta^{\prime }-\beta_{0}}{\Delta \beta }\right\rfloor . \end{array}$$

*E*(*λ*′, *β*′) can be calculated through bilinear interpolation:

(4) Eλ′,β′=1−p1−qEi,j +p1−qEi,j+1 +p1−qEi+1,j +pqEi+1,j+1,

where *p* and *q*, respectively, are

(5) $$\begin{array}{c} p=\lambda^{\prime }-i\times \Delta \lambda ,\;\;q=\beta^{\prime }-j\times \Delta \beta . \end{array}$$

**Figure 3:**
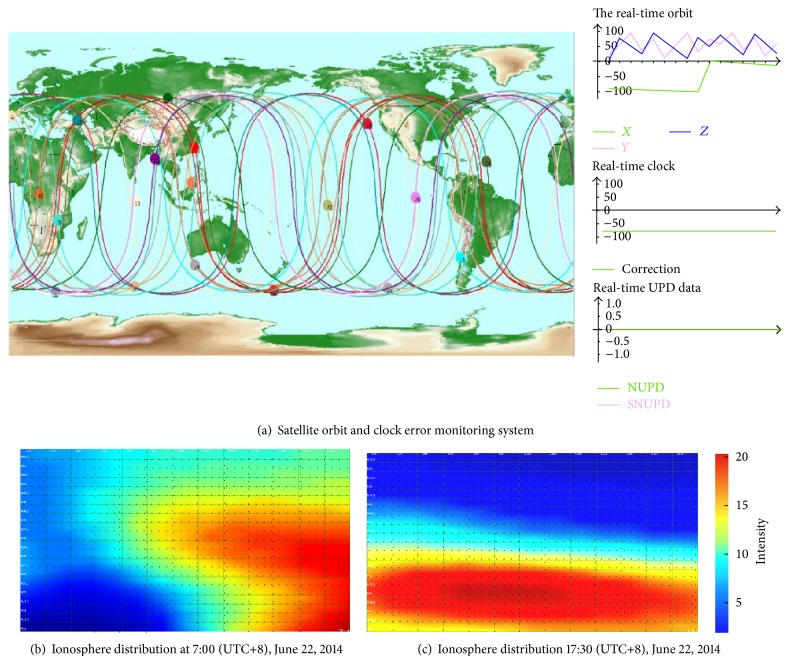
Ionosphere grid model and distribution.

The discrete grid point correction updates about every five minutes. In one period, the broadcast strategies of the hierarchy and increment plus the density are adopted to provide the ionospheric correction from thick to thin and from common to individual for the users based on their location. Satellite orbit clock error monitoring, the ionospheric grid model, and change distribution are shown in Figure 3.

### 4.3. Vehicle Terminal Design

**Figure 4:**
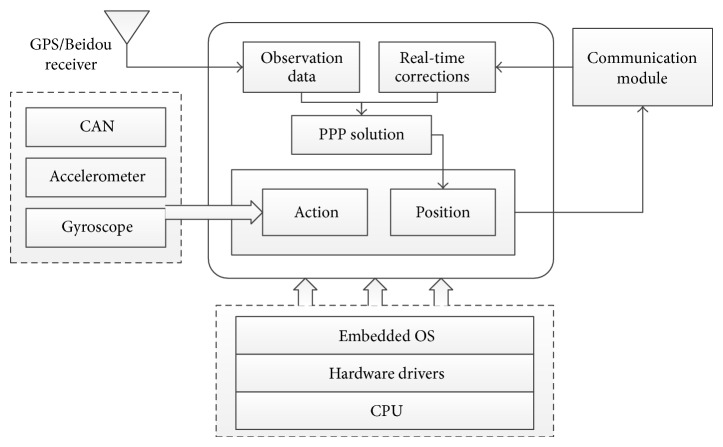
The structure of vehicle terminal.

The vehicle terminal includes data communication, navigation positioning, and status awareness, designed for human-computer interaction. The information terminal of the vehicle network receives the positioning corrections and service information broadcast by the server and simultaneously uploads the location and status data to the background. The terminal equipment carries the embedded operating system onto the main board and provides various pieces of equipment such as the ports of the GPS/BDS vehicle; controller area network (CAN); and 3G/WiFi. The terminal also realizes applications with the precise positioning processing of the upper layer and prewarning. The terminal architecture is shown in Figure 4.

The terminal uses a single-frequency GPS/BDS receiver and communication module to receive the orbit, clock error, and ionospheric correction broadcast by the background in real time. Combined with the satellite broadcast ephemeris, the precise satellite location and clock error of the satellite can be obtained. Furthermore, the position coordinates can be obtained through the resolve of the precise single-point positioning. Simultaneously, sensors such as the vehicle-used gyroscope and accelerometer access the vehicle CAN bus as part of the vehicle control information. The location and status data of the terminal are also sent to the background for monitoring and servicing of the vehicle network target.

## 5. Virtualization-Based Large-Scale Data Communication Architecture

The user population in the vehicle network is large and widely distributed. Data communication depends on the mobile Internet; thus it is easy for concurrent data communications to have an overly high load and a blocking node may influence communication reliability. If correction messages are used to serve the user population in the vehicle network, then a set of efficient and reliable communication architectures is needed. This section details the design for virtualization of large-scale data communication architecture in the mobile Internet environment. The main methods are as follows: first, we utilized a virtualized communication cluster, and designed a high-concurrency. Cluster-scheduling algorithm, which contribute to the “cloud computing” system for the precise positioning service; second, we designed an adaptive link maintenance protocol that helps to maintain the connection between the terminal and VCA.

### 5.1. VCA High-Concurrency Cluster-Scheduling Algorithm

The broadcast server is designed as a centralized control and distributed broadcast system. The system management node is used for short connections with small data, such as authentication management handshake communication and handshake steps, whereas the clustering VCA is responsible for the data flow broadcast. The management nodes include the authentication management module, resource allocation module, and resource monitor module, which are responsible for user authentication, resource allocation, and process status monitoring, respectively. According to the VCA load and communication protocols, we designed the resource allocation and scheduling (RAS) algorithm.

- The connections of the single VCA are limited, and the quantity of the VCA is dynamically adjusted. According to the user connections, the new VCA is opened while the free VCA is closed.
- According to the creation time and process load, the proper VCA is chosen to allocate a connection request.

The specific contents of the RAS algorithm are introduced as follows. The implementation is shown in Algorithm 1.

*Steps of RAS*. The set of all the VCAs in the system is noted as *UC* = {*C*_*i*_∣*i* = 1,2,…*M*}, where *M* represents the total number of processes. If the total connections load_*i*_ of the *C*_*i*_ communication reach the set threshold, add load_*i*_ into the full-load process queue *C*_*F*_ and add the left connections into the nonfull load process queue *C*_*U*_, which contains *N*_*U*_ processes. In the *UC*, the VCA first found by the VCA is noted as *C*_*S*_, and the connection of *C*_*S*_ is noted as *ns*. When the system is initialized, the number of processes is assumed to be *K*, and the minimum *N*_*U*_ is assumed to be *K*.

1. When the system is initialized, all the generated VCAs are added into the *C*_*U*_ and are randomly distributed to the arriving users. The connections of all the VCA in *C*_*U*_ are checked, and the VCAs that satisfy the full-load condition from the *C*_*F*_ are removed and then added into the *N*_*U*_ queue.
2. The number *C*_*U*_ of *C*_*U*_ is checked. If *N*_*U*_ < *K*, new VCAs *K* − *N*_*U*_ are created in the *C*_*U*_. If *N*_*U*_ > *K*, the connections of *C*_*S*_ in *C*_*F*_ are checked. If the connection is 0, then the *C*_*S*_ is closed.
3. For newly generated connection requests, a section of the VCA in the *C*_*U*_ is chosen to provide services. There are two selection strategies: the time priority strategy (TPS) selects the latest-created VCA in the *C*_*U*_, according to the sequence of the creation time, and the load priority strategy (LPS) utilizes the connections to express the load and select the VCA with the least connections in the *C*_*U*_. These two strategies have different utilizations of resources. We did the experiments described in Section 4.2 to compare the average number and utilization rate when using different strategies.
4. The allocated VCA is checked. If a full load is reached, this allocated VCA is removed from the *C*_*F*_ and then added to the *C*_*F*_ queue. Afterwards, step (2) is repeated.

### 5.2. Adaptive Maintenance of Communication Link

In the mobile Internet environment, the stability of the communications link is relatively poor and has packet loss and error codes. In addition, the rapid movement of the terminal influences data communications. Therefore, the system was designed with a set of adaptive communication link maintenance strategies to maintain reliable communications between the terminal and the VCA:

- Multisource and multiprotocol communication mode. The network layer combines UDP and TCP, thus providing multiple VCA service resources;
- dynamic maintenance of the links between the terminal and the VCA. According to the service quality, service resources are adaptively switched and dynamic control of the code rates and error correction code for the transferred data are executed.

**Figure 5:**
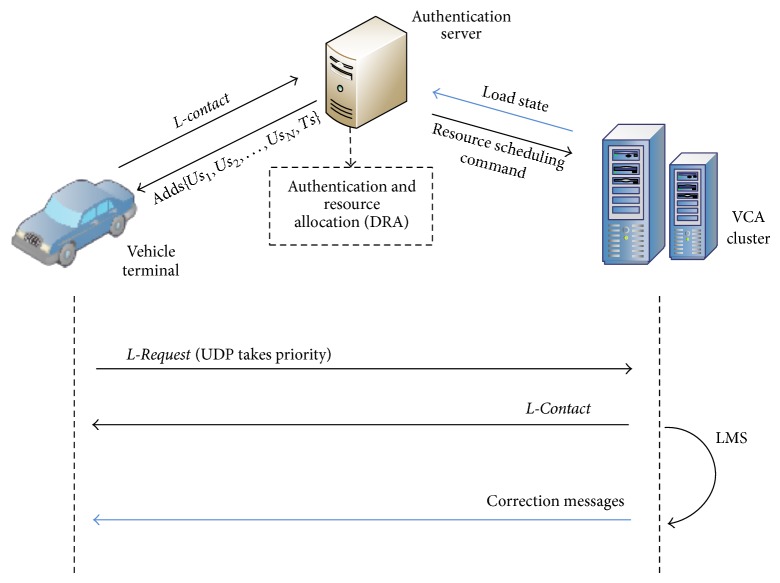
Communication protocol of the data broadcasting.

The maintenance strategy is introduced as follows. This strategy adopts the communication protocol design shown in Figure 5, including the following steps.

1. According to the known port address of the management nodes, the client-side establishes the TCP handshake L-*contact*, tests the link connectivity, and completes the authentication process.
2. After determining the client identification, the management nodes return to the address port sequence of a section of the communication process from the service queue *S*, Adds{*Us*_1_, *Us*_2_,…, *Us*_*N*_, *Ts*}, where *Us* represents the UDP process, *N* represents the number, *Ts* represents the TCP process, and the number is 1.

According to the address sequence, the terminal sends an* L-Request* to the VCA of the data broadcast cluster. If* L-Confirm* is received within the connection time limit, a long connection is established with the VCA. The UDP process in the address sequence will then be prioritized. If the terminal fails to connect, a sequence switch will be adopted and the TCP process will be used. If all fail to connect, step (1) is repeated.

A link maintenance strategy (LMS) is adopted to control both the terminal and the VCA: the terminal is noted as* TE* and the connected VCA in the terminal is noted as *C*_*T*_; the communication link between the* TE* and *C*_*T*_ is noted as *L*_*T*_(*TE*, *C*_*T*_); the time that the link is established is noted as *T*_*C*_; the waiting time limit of the* TE* data flow is noted as *t*_*r*_; the code rate of the data sent is noted as *B*_*T*_; the length of the error correcting code is noted as *Ec*; the bit error rate of the terminal receiving data is noted as *Ber*_*T*_; and the threshold of the bit error rate is noted as *ψ*.

A connection *L*_*T*_(*TE*, *C*_*T*_) is automatically reestablished when the IP address of the terminal changes. When the waiting time of the terminal is over, denoted as *t*_*r*_, the current link *L*_*T*_ will be cut off and a new connection will be established. If the receiving time of the* TE* on the bit error rate *Ber*_*T*_ is over *C*_*T*_, *C*_*T*_ will decrease the data transfer rate of *L*_*T*_ and the length of the error-correcting code will be added. The implementation is shown in Algorithm 2.

**Figure 6:**
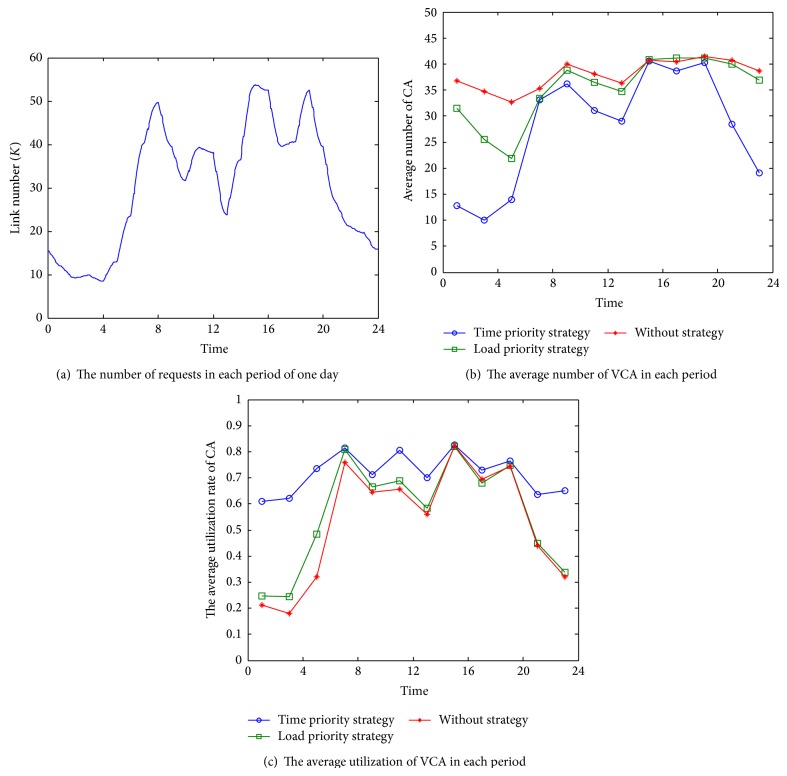
High-concurrency cluster-scheduling simulation.

A high-concurrency request simulation experiment was conducted on the strategies and algorithm in the proposed system; the validity of the system was verified. We simulated 100,000 daily user connection requests, and the working conditions of the system that adopted the scheduling algorithm were tested. The number of simultaneous online users in each period of one day is shown in Figure 6(a), and the peak concurrent request number is about 53 thousands.  Figure 6(b) shows the average VCA number of the background system in different time, and Figure 6(c) shows the corresponding average utilization of VCA. The red curve represents the result without using any scheduling strategy, the green curve is the result applied with the load priority strategy (LPS) while the blue is applied with the time priority strategy (TPS). As can be seen from the figure, the VCA number significantly decreases after applying a scheduling strategy to the background, which means the system process overhead will be less. VCA utilization is the ratio of a VCA's actual connection number and its maximum connection, and the utilization value reflects the efficiency of the system. The results shown in Figure 6(c) indicate that the average utilization of VCA improved with the scheduling strategy. Between the two strategies in the algorithm, the time priority strategy achieves less VCA average number and a better utilization. So, in Algorithm 1, the time priority strategy is more advantageous.

## 6. Verification of the Precise Positioning Vehicle Network Location Service and Design of Active Safety Service

To validate the system service, we conducted a long-term, across-Internet service provider, across-region comprehensive test, and analyzed the positioning accuracy in urban city. The outcome showed that this system could supply the lane-level location service. With the high precision positioning provided by this system, a typical active safety service can be designed. Precise space-time reference is the core factor to achieve active safety. This part focuses on the data accuracy verification of this vehicle network location system. And, based on this experiment's outcome, we also designed a typical vehicle active safety service in the situation of lane-level location.

### 6.1. Verification of the Positioning System Effect

The allocation of the system platform in this paper adopted the Alibaba cloud computing server, whose virtualization scheduling advantages avoid the network bottlenecks caused by different network operators. To verify the positioning precision and service quality in an urban environment, vehicle tests were conducted in Wuhan, Shanghai, Zhongshan, and Beijing in June 2013.

**Figure 7:**
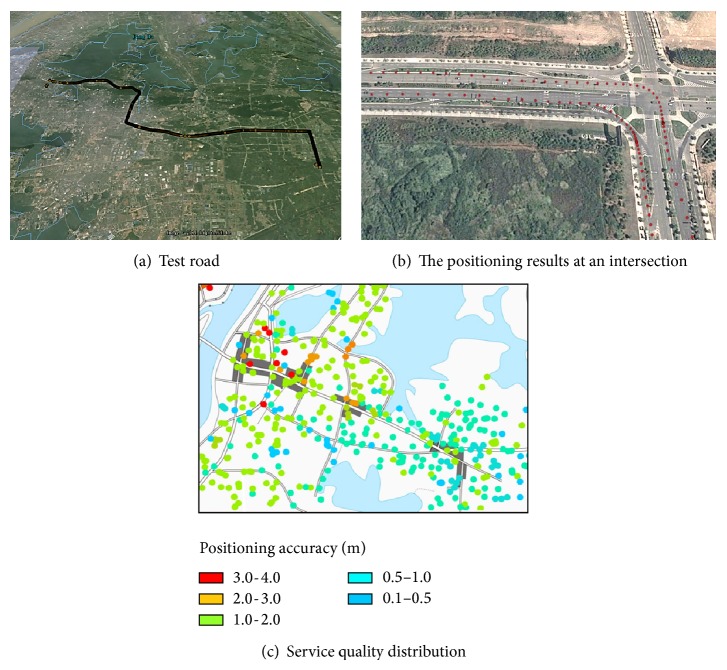
Verification of the positioning service.

Figure 7 shows the results of the vehicle-positioning test in Wuhan. We conducted the round-trip tests on an urban main road from Wuhan University to the Optical Valley 7th Road.  Figure 7(a) is a general view of the test roads.  Figure 7(b) shows the positioning results generated by our positioning system at a certain intersection of the test roads. As we see in Figure 7(b), red dots are the trajectories of a car, from which we can clearly distinguish the lane in which the car was driving. And, through the whole test section, the positioning terminal can obtain a continuous and stable real-time positioning result.

A heat map of the whole positioning service quality in the urban area shown in Figure 7(c) was created using the long-term test results from numerous vehicles. The areas in this figure are Wuchang district in Wuhan. The dark regions are downtown areas with crowded high-rises. We use dots with different colors to express the average positioning accuracy in different areas and the value of the color is presented in the legend. As can be seen, in most of the test areas we can obtain a positioning accuracy within 2 meters, and, in some area far away from downtown, the average accuracy can be 0.5 meters and even higher. A few areas with poor positioning precision are located mostly in the commercial districts with high-rise buildings or intersections of highways (usually with viaducts). Satellite signals in these areas are blocked out, and thus the available satellites decrease greatly, further causing imprecise positioning, which is a congenital defect of satellite positioning. Test results indicate that the precise positioning service proposed in this paper can achieve a positioning accuracy of submeter level in most city environment, and the lane cars driving on can be distinguished from this information. Thus, this vehicle network system can satisfy a lane-level location service for drivers, for example, distinguishing whether the vehicle is on the main road or on the side road, as well as the accurate position and time of the entry-and-exit ramp entrance.

### 6.2. Design of the Lane-Level Active Safety Service

The system provides a lane-level precise positioning service for an urban area. The vehicle network platform can realize the perception and tracking of vehicle behaviors by monitoring a vehicle track and identifying vehicular movements, such as illegal lane changes and dangerous turning, to identify road hazards.

**Figure 8:**
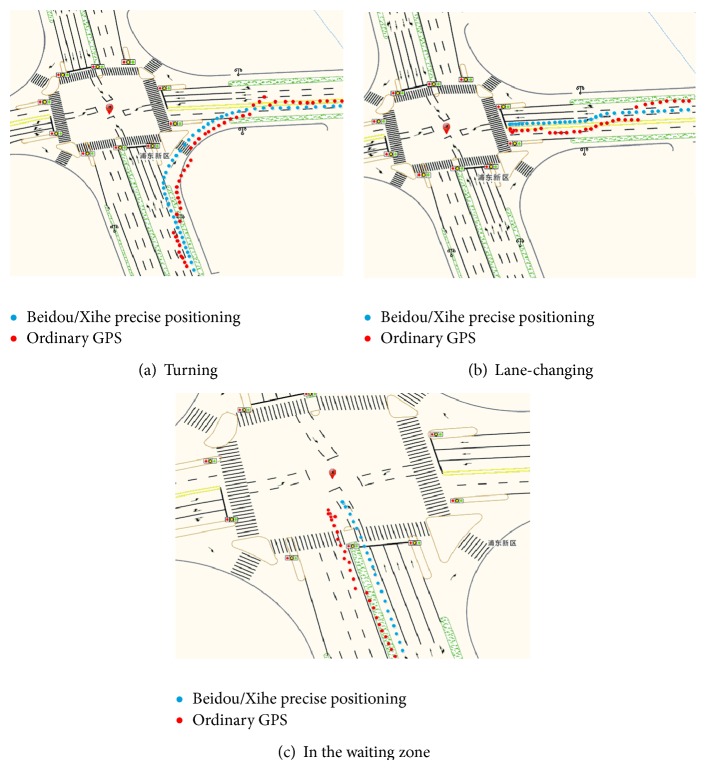
Vehicle movement test.

This study deployed this precise positioning service platform to conduct a monitoring experiment on the lane-level vehicle behaviors on urban online vehicles by combining the traffic management systems of Shanghai, Zhongshan, and other cities; Figure 8 shows the test result of the junction between Chengshan Road and Jinxiu Road, in Pudong District, Shanghai. The test was conducted on May 14, 2014. The experimental vehicles adopted the NovAtel GPS board card. Through the WCDMA 3G network, the correction message was received. The accurate positioning coordinate was calculated with the output frequency that ranged from 1 Hz to 5 Hz and was then matched with a high-precision map.

The red dots in the figure are the positioning results from ordinary GPS, while the blue are from the Beidou-Xihe high-precision positioning. The output results indicate that when the vehicles were normally driving on the urban main road (with an average experimental speed of 60 km/h), the monitoring platform with high-precision positioning could clearly distinguish the vehicle lanes and determine vehicular movements, such as lane changing and turning, while those with ordinary GPS do not have these abilities. Based on precise positioning, the vehicle network in this study also perceived the ability of dangerous movements of the vehicles in real time. Such perception provided the conditions for the construction of an active vehicle safety service. In this vehicle network platform, we designed the service model based on the dynamic geofence. The service model can deliver an active information push for dynamic danger targets for risk control in the intelligent transportation system field.

A traditional vehicle network is efficient in actively pushing static hidden risk information of an accident-prone area to relevant vehicles through static geofence [16] technology. These observations however are insufficient to satisfy the demands for control of hidden dangers. Data indicate that over 60% of traffic accidents relate to passenger vehicles, such as school buses, as well as to heavy freight and dangerous chemical transportation vehicles. In a vehicle network environment, the transport conditions of these dynamic danger targets can be rapidly and accurately pushed into the surrounding relevant vehicles, which can then improve the cognition of the front (future) dynamic safety risk. Consequently, we set up dynamic geofence centering on the mobile vehicles. This network can process real-time coordination of multiple moving targets, recognizing the latent risk targets sending this information to cars around this dangerous vehicle.

**Figure 9:**
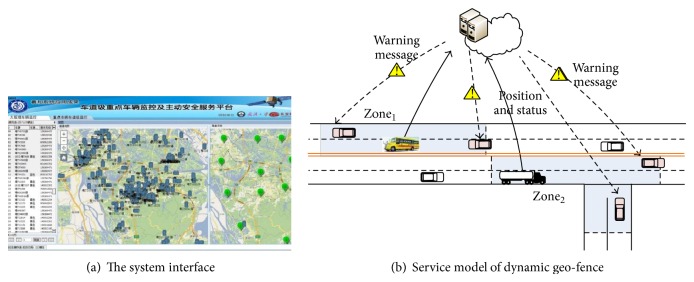
Traffic management platform based on dynamic geo-fence.

Through the active coordination and information about multiple dynamic danger targets, the following two aspects of traffic safety scenes can be efficiently dealt with: (1) when a high-danger target (e.g., dangerous chemical transportation vehicles) occurs in front, a prewarning can be sent to the driver. This kind of prewarning information can be used to decrease the accident risk in certain situations, such as long-distance night driving or in relation to hidden dangers. (2) When a high-right-of-way vehicle (e.g., school bus) is in front of a driver, the risk of a right-of-way conflict of the surrounding vehicles can be decreased. We used this model as the plugin for the real-time monitoring platform in the proposed traffic management vehicle network and in the experiments conducted on the Traffic Management Platform (copy) of Zhongshan, Guangdong.  Figure 9(b) shows the service model, zone_1_ is the geofence area of a school bus at a particular time, and zone_2_ is the geofence area of a truck. The position and shape of the geo-fence area is related with the target's movement and status, and vehicles in the area will get a warning message of danger.

## 7. Conclusion

The ground based augmentation system used in the survey field was redesigned to meet the high precise positioning requirement of large-scale vehicle network. Based on this a precise positioning vehicle network location service platform is realized. In which the wide-area GPS/BDS satellite navigation and positioning system were applied to the vehicle network system to solve the problems of a traditional vehicle network, due to lack of uniform and high-quality space-time information. Simultaneously, this paper demonstrates that through a precise positioning service, a dynamic geofence-based information push service can provide an efficient and reliable active safety service for a vehicle network.

Verifications through experiments reveal that this vehicle network system can provide a continuous and stable real-time precise positioning service for numerous users in most urban cities. Through the lane-level vehicle track monitoring, road danger targets can be identified and an active safety service can be constructed. Relevant achievements were included in the national BeiDou-Xihe research project, the basic foundation for the construction of an intelligent city. However, in some special situations for instance, cars move too fast and the satellite signals are blocked out; this system still has the possibility of imprecise positioning. Besides, in order to perceive the activity of the moving vehicles' dangerous movements, more factors need to be considered. We will solve these problems in our later work.

## Figures and Tables

**Algorithm 1:**
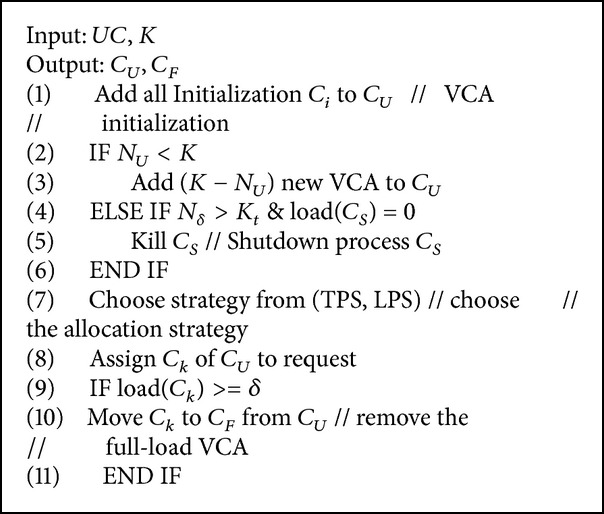
Resource allocation and scheduling.

**Algorithm 2:**
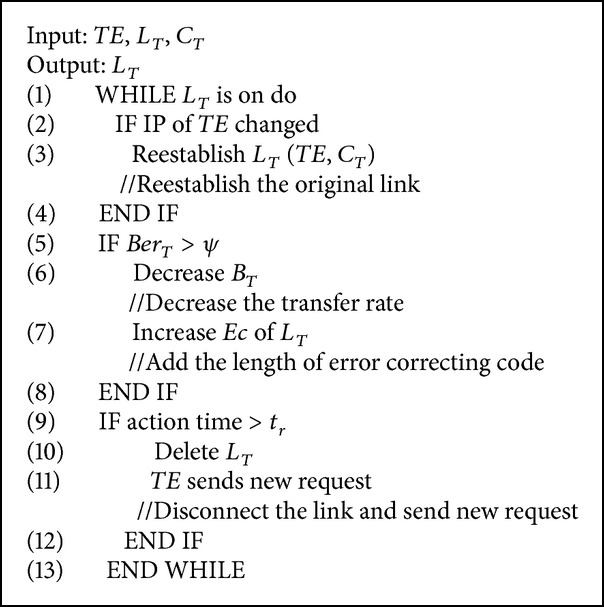
Link maintenance strategy.
